# CDK9 inhibitor elicits APC through a synthetic lethal effect in colorectal cancer cells

**DOI:** 10.1016/j.gendis.2024.101220

**Published:** 2024-01-24

**Authors:** Tingming Liang, Lulu Luo, Xinru Xu, Yajing Du, Xinbing Yang, Jiahui Xiao, Xiaoyan Huang, Huiying Yang, Siyu Wang, Li Guo

**Affiliations:** a Jiangsu Key Laboratory for Molecular and Medical Biotechnology, School of Life Science, Nanjing Normal University, Nanjing, Jiangsu 210023, China; b State Key Laboratory of Organic Electronics and Information Displays & Institute of Advanced Materials (IAM), Nanjing University of Posts & Telecommunications, Nanjing, Jiangsu 210023, China

Colorectal cancer (CRC) is a significant global health concern, ranking third in incidence and second in mortality among all cancer-related diseases.^1^ Therefore, it is imperative to prioritize research efforts focused on CRC prevention and treatment to effectively reduce the associated incidence and mortality rates. Synthetic lethality (SL) refers to the phenomenon in which cells harboring two specific gene mutations simultaneously undergo cell death, whereas cells with a single mutation in either of the genes can survive normally. SL strategies are widely concerned in personalized cancer treatment, with the application of PARP inhibitors marking a significant milestone.^2^

The adenomatous polyposis coli (APC) gene demonstrates a high mutation rate of up to 60% in CRC, rendering it a crucial molecular target in investigating SL anti-cancer strategies. The selection of APC and its SL gene partners has been proven valuable for studying SL gene pairs in CRC. To address APC-related SL-based genetic interactions in CRC, SLOAD, a synthetic lethal gene prediction platform (http://www.tmliang.cn/SLOAD/),^3^ was used to screen APC-related interactions, and APC and cyclin-dependent kinase 9 (CDK9) were finally selected as a candidate gene pair with promising clinical application in personalized medicine. To verify the SL effect of APC and CDK9, further experimental validation was conducted to elucidate its role in cancer cell proliferation and cell death.

Initially, three distinct CRC cell lines expressing APC were employed to investigate the SL effect between APC and CDK9. HCT116 cells represented APC wild-type cells capable of expressing a 312-KDa protein, termed WT-HCT116. The KO-HCT116 cell line denoted APC knockout cells engineered in our laboratory using CRISPR/Cas9 technology. Meanwhile, the SW480 and HT29 cell lines carried mutated APC genes, referred to as Mut-SW480 and Mut-HT29, respectively. We utilized LDC000067, a specific CDK9 inhibitor, to assess cell viability through the CCK8 assay. Within 24 h of LDC000067 treatment, a significant reduction in viable cells was evident in the KO-HCT116, Mut-SW480, and Mut-HT29 cells (Fig. 1A–C). The colony formation assay revealed a marked decrease in cell proliferation among the KO-HCT116 and Mut-SW480 cells following exposure to LDC000067 (Fig. 1D). Owing to the distinct gene mutations in each cell line, the sensitivity to drugs varied. These findings provided preliminary evidence of an SL interaction between APC and CDK9, suggesting heightened sensitivity of APC-deficient cells to CDK9 inhibitors. In addition, we conducted 5-ethynyl-2′-deoxyuridine (EdU) staining to evaluate CRC cell proliferation with three different APC mutations after treatment with APC inhibitors (0, 2, and 4 μM). The results indicated a stronger basal cell proliferation ability in the KO-HCT116 and Mut-SW480 cells than in the WT-HCT116 cells. Treatment with APC inhibitors markedly suppressed the proliferation of KO-HCT116 and Mut-SW480 cells in comparison to the WT-HCT116 cells (Fig. 1E, F), consistent with the CCK8 assay results.

**Figure 1:**
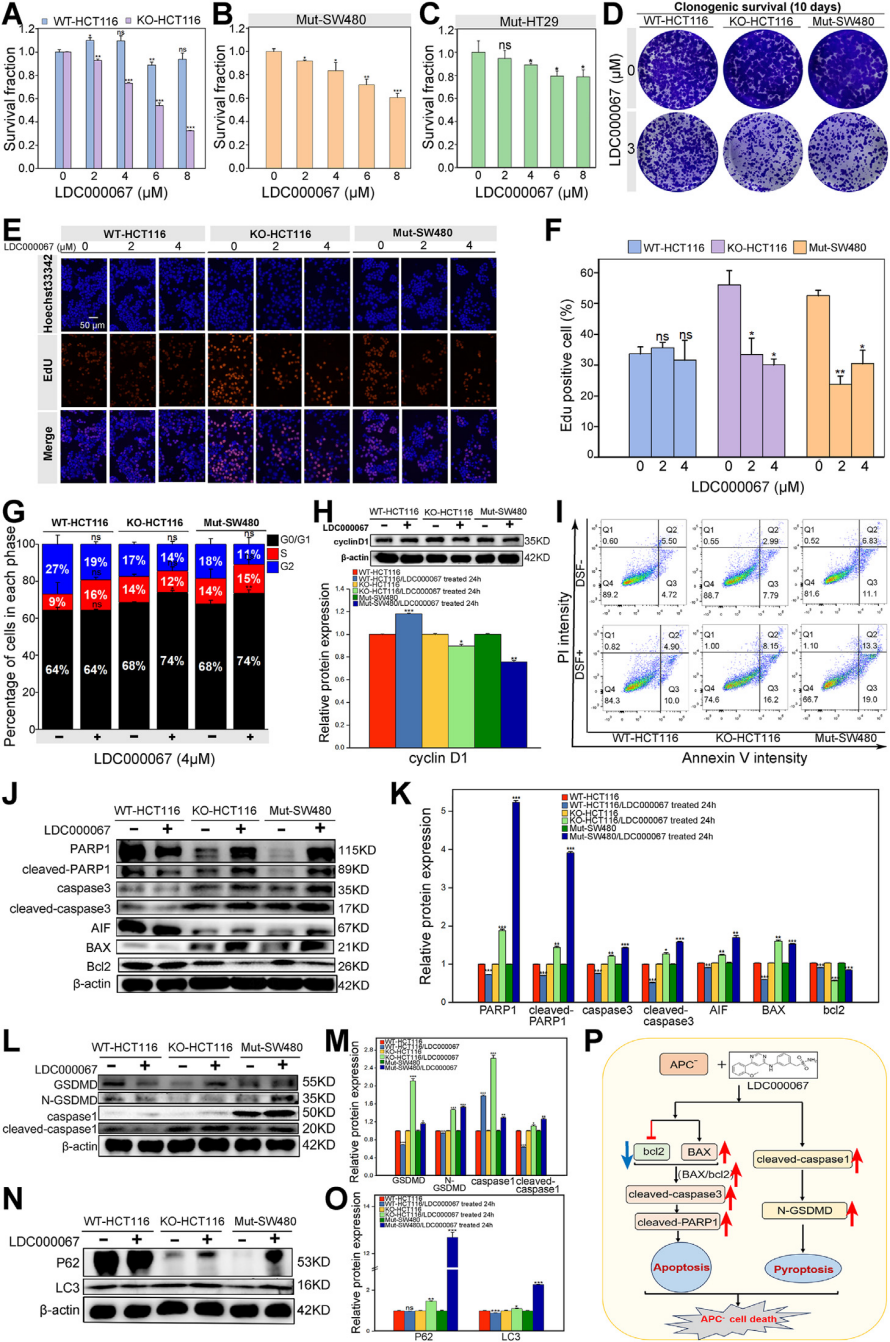
The molecular mechanism underlying the synthetic lethality induced by a CDK9 inhibitor in APC-deficient colorectal cancer cells. **(A)** The effects of LDC000067 treatment on WT-HCT116 and KO-HCT116 cells were investigated by assessing cell viability and constructing histograms. The results demonstrated a reduction in the survival rates of the KO-HCT116 cells following LDC000067 treatment, whereas the WT-HCT116 cells exhibited no significant changes in response to LDC000067. **(B)** Cell viability was evaluated by treating Mut-SW480 cells with varying concentrations of LDC000067, and histograms were generated accordingly. The results demonstrated a dose-dependent decrease in cell viability, with increasing concentrations of LDC000067. **(C)** Cell viability was evaluated by treating the Mut-HT29 cells with varying concentrations of LDC000067, and histograms were generated accordingly. The results demonstrated a dose-dependent decrease in cell viability, with increasing concentrations of LDC000067. **(D)** Colony formation assay was performed, wherein 2000 cells were plated in a 12-well plate and compared with WT-HCT116 cells. The results indicated a substantial impairment in the proliferation ability of the KO-HCT116 and Mut-SW480 cells after LDC000067 treatment. **(E)** The proliferation of CRC cells with three different APC mutations after treatment with APC inhibitors was detected using EdU staining. **(F)** Statistical analysis of the EdU-positive cells in representative images according to C. **(G)** The cell cycle analysis results were organized into tables and presented as histograms using Origin software. The histograms depict G0/G1 phase arrest in the KO-HCT116 and Mut-SW480 cells following LDC000067 treatment. **(H)** After 24-h treatment with LDC000067, a decrease in cyclin D1 expression was observed in the KO-HCT116 and Mut-SW480 cells, indicating a reduction in the number of G0/G1 phase cells transitioning to the S phase. Additionally, an increase in the proportion of cells in the G1 phase suggested cell cycle arrest at the G1 phase (upper panel). The grey analysis histogram of cyclin D1 expression is also presented (lower panel). **(I)** Apoptosis was assessed using flow cytometry, and scatter plot analysis was performed using FlowJO software, revealing a higher apoptotic rate in the KO-HCT116 and Mut-SW480 cells compared with that in the WT-HCT116 cells after LDC000067 treatment. **(J)** Following a 24-h treatment with LDC000067, the expression of the anti-apoptotic protein Bcl2 was reduced in the KO-HCT116 and Mut-SW480 cells, whereas the expression of the pro-apoptotic protein BAX was up-regulated. This phenomenon resulted in an increased BAX/Bcl2 ratio, which in turn activated the apoptosis system. Moreover, elevated levels of cleaved caspase 3 and cleaved PARP1 were observed, suggesting the involvement of CDK9 inhibitors in the apoptosis process following treatment of the APC-deficient colorectal cancer cells. **(K)** The grey analysis histogram of apoptosis-related proteins corroborated the aforementioned findings, illustrating the changes in the expression levels of proteins associated with apoptosis. **(L)** Following a 24-h treatment with LDC000067, the expression of caspase1 and GSDMD was up-regulated in the KO-HCT116 and Mut-SW480 cells, indicating an augmentation of pyroptosis mediated by caspase1. **(M)** The grayscale analysis histogram of cell pyroptosis-related proteins corroborates the aforementioned results. **(N)** After 24-h treatment with LDC000067 in the WT-HCT116, KO-HCT116, and Mut-SW480 cells, the expression of the autophagy marker protein LC3 and the autophagy substrate P62 was up-regulated in the KO-HCT116 and Mut-SW480 cells, suggesting that there was no increase in autophagy. **(O)** The grayscale analysis histogram of the autophagy-related proteins supports the aforementioned results. All experiments were repeated three times independently, and the data were statistically analyzed using a *t*-test. **(P)** APC-deficient colorectal cancer cells exhibit activation of the apoptosis and pyroptosis systems following treatment with the CDK9 inhibitor LDC000067, resulting in cell death (by FigDraw). Treatment with LDC000067 in the KO-HCT116 and Mut-SW480 cells resulted in decreased expression of the anti-apoptotic protein Bcl2 and increased expression of the pro-apoptotic protein BAX, leading to an elevated BAX/Bcl2 ratio and apoptotic pathway initiation. The up-regulation of cleaved caspase-3 and cleaved-PARP1 expression further contributed to the apoptotic process. Additionally, marked up-regulation of the pyroptosis markers cleaved-caspase1 and N-GSDMD indicated the activation of the caspase1-mediated pyroptosis pathway. The concurrent increases in the apoptosis and pyroptosis systems were responsible for the death of APC-deficient colorectal cancer cells treated with CDK9 inhibitors. ∗*P* < 0.05, ∗∗*P* < 0.01, ∗∗∗*P* < 0.001.

To delve deeper into the diminished proliferation of APC-deficient cells upon LDC000067 treatment, we evaluated the impact of CDK9 inhibitors on cell cycle progression. Flow cytometry analysis was performed on the WT-HCT116, KO-HCT116, and Mut-SW480 cells following a 24-h exposure to 4 μM LDC000067. The results revealed a notable arrest in the G1 phase in both the KO-HCT116 and Mut-SW480 cells compared with that in the WT-HCT116 cells (Fig. 1G). These findings suggest that CDK9 inhibitors induced G1 phase arrest specifically in APC-deficient cells, shedding light on the mechanism underlying the observed reduction in proliferation in these cellular contexts. Western blotting demonstrated a marked reduction in β-catenin (Fig. S1) and cyclin D1 expression (Fig. 1H) in both the KO-HCT116 and Mut-SW480 cells compared with the WT-HCT116 cells. These outcomes support the hypothesis that the combined effect of APC deletion and CDK9 inhibition contributed to increased cell death and reduced proliferation. The down-regulation of cyclin D1 in APC-deficient cells following CDK9 inhibitor treatment suggests the potential role of cyclin D1 in mediating these effects. In summary, our results suggest that the decreased proliferation observed in APC-deficient cells after CDK9 inhibitor treatment can be attributed to the alteration of cell cycle progression induced by the inhibitor. Furthermore, this treatment increased cell death and reduced proliferation, facilitated by cyclin D1 down-regulation.

After treatment with CDK9 inhibitors, a marked decline in cell viability was evident in the APC-deficient cells. To attain a comprehensive understanding of this phenomenon, we investigated three well-established mechanisms of cell death, apoptosis, pyroptosis, and autophagy. Our initial focus was on unraveling the potential involvement of apoptosis in the synergistic lethal effect associated with APC and CDK9. Flow cytometric analysis unveiled a substantial increase in apoptosis in both the KO-HCT116 and Mut-SW480 cells following 24 h of LDC000067 treatment (Fig. 1I; Fig. S2), and a down-regulation of the apoptosis-inhibitory protein Bcl2 was observed. In contrast, there was an up-regulation in the expression of the apoptosis-promoting protein BAX, resulting in an elevated BAX/Bcl2 ratio. This modulation signaled the activation of the apoptotic signal transduction pathway. Moreover, cleaved-caspase3 expression was up-regulated, resulting in increased AIF and cleaved-PARP1 expression (Fig. 1J, K). These results indicate a substantial surge in apoptosis within APC-deficient cells after treatment with CDK9 inhibitors, underscoring the pivotal role of apoptosis as a mechanism contributing to the observed cell death in APC-deficient cells upon treatment with CDK9 inhibitors.

Pyroptosis has garnered attention in the context of the tumor microenvironment and is implicated in colitis-related cancers.^4^ Western blotting revealed a notable up-regulation in the expression of pyroptosis markers, specifically cleaved-caspase1 and N-GSDMD, after a 24-h treatment with 6 μM LDC000067 in the KO-HCT116 and Mut-SW480 cells compared with that in the WT-HCT116 cells (Fig. 1L, M), further highlighting the role of pyroptotic cell death as a potential mechanism. Autophagy has been implicated in colon cancer development, particularly concerning cancer stem cells. Reducing CDK9 transcription may lead to a decrease in p62 expression,^5^ and therefore simultaneous inhibition of both APC and CDK9 necessitates an investigation into their impact on autophagy. Following a 24-h treatment with 6 μM LDC000067, the expression of the autophagy marker LC3 and the autophagy substrate protein p62 was up-regulated in the KO-HCT116 and Mut-SW480 cells (Fig. 1N, O), suggesting that CDK9 inhibitor treatment initiated autophagy. However, it appears that the autophagic process may not progress smoothly and can encounter blocks. Remarkably, following treatment of APC-deficient CRC cells with CDK9 inhibitors, there was a significant increase in pyroptosis, whereas a similar increase in autophagy was not observed. These observations indicate that increased apoptosis and pyroptosis represent the primary mechanisms underlying cell death resulting from the simultaneous inhibition of APC and CDK9.

After treatment with the CDK9 inhibitor LDC000067, minimal changes were observed in the WT-HCT116 cells, while the KO-HCT116 and Mut-SW480 cells exhibited increased cell death and reduced cell proliferation, implying that the heightened apoptosis and caspase1-mediated pyroptosis pathways contributed to increased cell death. Furthermore, the accumulation of cells in the G1 phase resulted in cell cycle arrest, ultimately leading to diminished cell proliferation. Consequently, CDK9 inhibitor treatment resulted in escalated SL and decreased cell proliferation in the APC-deficient CRC cells, facilitating the targeted elimination of CRC cells at the cellular level (Fig. 1P). Taken together, our study elucidates the molecular mechanism underlying CDK9 inhibitor-induced SL in APC-deficient cells, providing novel insights into CRC pathogenesis and the development of innovative treatment strategies.

## Author contributions

TL and LG conceptualized and designed the project; LL, XX, YD, YY, JX, XH, HY, and SW performed experiments and data analysis; TL, LL, and LG wrote the manuscript; all authors read and approved the manuscript.

## Conflict of interests

The authors declared no conflict of interests.

## Funding

This work was supported by National Natural Science Foundation of China (No. 62171236), the key project of social development in Jiangsu Province, China (No. BE2022799), the key projects of Natural Science Research in Universities of Jiangsu Province, China (No. 22KJA180006), the Open Research Fund of State Key Laboratory of Bioelectronics, Southeast University (No. SKLB2022-K03), and the Priority Academic Program Development of Jiangsu Higher Education Institution (PAPD) (China).

## Data availability

The data that support the findings of this study are available from the corresponding author upon reasonable request.

## Figures and Tables

**Figure S1:**
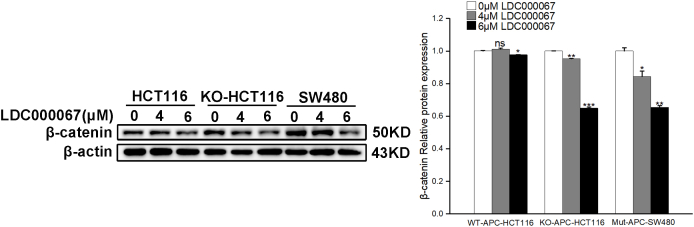


**Figure S2:**
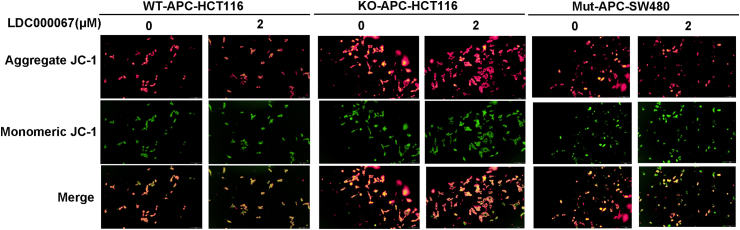


## Appendix A Supplementary data

The following are the Spreprint:uupplementary data to this article:
