# Supplementary material for: CDK9 inhibitor elicits APC through a synthetic lethal effect in colorectal cancer cells
Source: Genes Dis. 2024 Jan 24;12(1):101220. doi: 10.1016/j.gendis.2024.101220 (PMC11472088; doi:10.1016/j.gendis.2024.101220)
Supplement: Multimedia component 1 [file mmc1.docx]

**Materials and methods**

**Data resources**

Following this, we employed the synthetic lethal gene prediction platform known as SLOAD (<http://www.tmliang.cn/SLOAD/>) to screen for genes that potentially exhibit a synthetic lethal pairing relationship with APC. Subsequently, we leveraged the high-throughput sequencing data available in the Cancer Genome Atlas (TCGA) to perform an analysis of the survival curve associated with APC.

**Cell culture**

To determine the synthetic lethal function of the selected genes, we utilized specific human colorectal cancer cell lines, namely WT-HCT116, KO-HCT116, and Mut-SW480. Among these cell lines, KO-HCT116 was generated through stable transfection using CRISPR/Cas9 technology to knock out the APC gene. Mut-SW480, on the other hand, is a human colorectal cancer cell line that naturally carries a mutation in the APC gene. All the cell lines were maintained in a controlled environment at 37 °C in a CO_2_ incubator. The culture medium used for these cell lines was DMEM high glucose medium (Keygen Biotech, China), supplemented with 10% fetal bovine serum (FBS, BRL-GIBCO, CA, USA).

**Cell Counting Kit-8(CCK8) experiment**

We employed the CCK-8 assay (APExBIO, Houston, USA) to assess the impact of different drug concentrations on cell viability. Initially, we prepared cell suspensions of WT-HCT116, KO-HCT116, Mut-SW480 and Mut-HT29 at a dilution of 8 × 10^4^ cells/mL. Subsequently, we added 8 × 10^3^ cells to each well of a 96-well plate, and after 24 hours of culture, the drugs were introduced. Following a 24-hour incubation period post-drug treatment, the CCK-8 assay was conducted. The procedures were carried out in a light-protected environment, and a CCK-8 diluent was prepared accordingly. The 96-well plates, containing the CCK-8 reagent, were covered with foil and placed in an incubator for 1 hour. Subsequently, the absorbance was measured at 450 nm using a microplate reader. Once the measurement was completed, the data was saved for further analysis. The survival rate was calculated using the formula: survival rate = (experimental group absorbance - blank group absorbance) / (control group absorbance - blank group absorbance).

**Cell clone survival experiment**

To investigate the synthetic lethal effect, we conducted cell clone survival experiments. Initially, we prepared suspensions of WT-HCT116, KO-HCT116, and Mut-SW480 cells and diluted them to a concentration of 8 × 10^4^ cells/mL. Subsequently, we seeded 2 × 10^3^ cells in each well of a 12-well plate. Following 3 days of culture, we added the respective drugs and placed the plate in an incubator for approximately 10 days. Upon completion of the cell culture period, we fixed the cells using a solution of 4% paraformaldehyde (Biosharp, China) and stained them with crystal violet for 15-20 minutes. After staining, we carefully washed the plate three times with PBS to remove any background staining. Subsequently, we removed the lid of the 12-well plate and inverted it onto a white paper surface for photography.

**Detection of CRC cell proliferation via 5-Ethynyl-2’-deoxyuridine (EdU) staining and hoechst 33342 staining**

The proliferation of CRC cells was evaluated using the Edu detection kit, which labels DNA synthesis by incorporating Edu. A working solution (10 μM) of Edu (100 μL) was added to CRC cells in a 96-well plate and incubated for 2 hours. The cells were then fixed in 4% paraformaldehyde for 15 minutes, sealed in phosphate-buffered saline (PBS) solution containing 3% bovine serum albumin (BSA), permeabilized in 0.3% Triton X-100 solution for 15 minutes, and washed with PBS (5 minutes per wash). Additionally, CRC cells in the 96-well plate were fixed with 4% paraformaldehyde and then permeabilized with 0.3% Triton X-100 solution for 15 minutes. They were then reacted with Hoechst 33342 staining solution (1 μg/mL) for 15 minutes, washed with PBS solution, and observed under a microscope for staining.

**Antibodies and reagents**

Primary antibodies including APC, Caspase3, Caspase1, P62, LC3, GSDMD, AIF, Bcl-2, BAX, PARP1, β-actin, Cyclin D1, were all purchased from Proteintech (Rosemont, IL, USA). Secondary antibodies, HRP goat anti-rabbit IgG(H+L) and HRP goat anti-mouse IgG(H+L), were also purchased from Proteintech (Rosemont, IL, USA). We purchase CDK9 inhibitor LDC00067 from MCE Corporation (New Jersey, USA).

**Western blot experimen****t**

In the drug-treated and untreated cell samples, 120 μL of RIPA protein lysate (Beyotime, China) was added to each well of a six-well plate. The RIPA-cell mixture was subjected to three freeze-thaw cycles. Subsequently, the mixture was centrifuged at 13,000 rpm for 10 minutes at 4 °C, and the supernatant was collected. To obtain a final concentration of 1×, 5× sample buffer (APExBIO, Houston, USA) was added to the supernatant. The mixture was then placed in a metal bath and heated at 100 °C for 7 minutes to extract the proteins, and store the extracted protein in a refrigerator at -80℃.

For SDS-PAGE gel electrophoresis, the protein Marker and the protein samples were loaded into the gel wells at a constant voltage of 120V. The electrophoresis time was determined based on the molecular weight of the target protein. Following electrophoresis, the protein bands in the gel were transferred onto a PVDF membrane (Millipore, the US). The transferred membrane was subsequently blocked with 5% skim milk for 1 hour. After blocking, the corresponding primary antibody solution was added and incubated overnight at 4 °C on a low-speed shaker. On the following day, the PVDF membrane was washed with TBST buffer (Servicebio, China) at room temperature and then incubated with an appropriate secondary antibody solution for 1 hour at room temperature. After washing the membrane with TBST, an ECL (Tianneng, China) color development solution was prepared. The PVDF membrane was immersed in the solution and exposed using a Tanon5200 scanner. Photographs were taken, saved, and grayscale analysis was performed using Image J software to generate a histogram of protein expression.

**Flow cytometry to detect cell apoptosis experiment**

To investigate apoptosis, we cultured WT-HCT116, KO-HCT116, and Mut-SW480 cells and passaged them into 6-well plates. Subsequently, the cells were treated with serum-free medium containing LDC000067, while serum-free medium containing an equivalent amount of DMSO was employed as a blank control. After 24 hours of incubation, both the treated cells and the supernatant were collected and prepared as a single-cell suspension. Then, the cells were stained with PI and Annexin V (apoptosis kit of Vazyme, China). Within 1 hour, flow cytometry was employed to detect the stained cells. The obtained experimental results were analyzed using Flwojo software, and a scatter plot representing apoptosis was generated. The apoptosis of APC-deficient cells were detected using the JC-1 detection kit according to the instruction manual (Vazyme, China).

**Flow cytometry to detect cell cycle experiment**

To investigate the cell cycle, we cultured WT-HCT116, KO-HCT116, and Mut-SW480 cells until they reached an optimal state. The cells were then treated with serum-containing medium containing LDC000067, while serum-containing medium with an equivalent volume of DMSO was used as a blank control. After 24 hours of culture, the treated cells and the supernatant were collected. Subsequently, the cell concentration was adjusted to 1 × 10^6^ cells/mL, and 1 mL of single cell suspension was obtained. Following centrifugation, the supernatant was discarded, and 500 μL of 70% cold ethanol was added to fix the cells. They were then stored at -20°C overnight. The cells that were fixed overnight were washed thrice with PBS, and the cell pellet was retained. Next, a pre-prepared staining solution containing 500 μL of PI/RNase A (cycle kit of Keygen Biotech, China) from Keygen's cycle kit was added to the cell pellet, and incubation was carried out at room temperature in the dark for 30 minutes. Within 1 hour of staining, flow cytometry was employed for detection. The experimental results were analyzed using Modfit software, and the cell cycle histogram was generated using Origin software.

**Statistical analysis of data**

All experimental data are expressed as mean standard deviation. Two-tailed T-Test was used to analyze the difference between the two samples, and One Way ANOVA was used to compare the two groups. P<0.05 means significant difference (*/#), p<0.01 means significant difference (* */# #), and p<0.001 means extremely significant difference (* * */# #).

**Figure Legends**

**Figure S1** The protein expression of β-catenin was significantly reduced in the APC-deficient cells upon treatment with CDK9 inhibitor (LDC000067).

**Figure S2** APC-deficient cells primarily undergo apoptosis when treated with the CDK9 inhibitor (LDC000067) using the JC-1 detection kit. The green fluorescence significantly increased in the drug treatment group. The green fluorescence represents apoptotic cells, whereas the red fluorescence represents living cells.
